# Prognostic Value of Estimated Glucose Disposal Rate and Systemic Immune-Inflammation Index in Non-Diabetic Patients Undergoing PCI for Chronic Total Occlusion

**DOI:** 10.3390/jcdd11090261

**Published:** 2024-08-26

**Authors:** Wenjie Chen, Yiming Liu, Yuchen Shi, Jinghua Liu

**Affiliations:** Center for Coronary Artery Disease (CCAD), Beijing Anzhen Hospital, Capital Medical University, Beijing Institute of Heart, Lung and Blood Vessel Diseases, 2 Anzhen Road, Chaoyang District, Beijing 100029, China; wenjiechen@mail.ccmu.edu.cn (W.C.); liuyiming202304@163.com (Y.L.)

**Keywords:** chronic total occlusion (CTO), percutaneous coronary intervention (PCI), estimated glucose disposal rate (eGDR), systemic immune-inflammation index (SII), major adverse cardiovascular events (MACEs)

## Abstract

Background and Objectives: Chronic total occlusion (CTO) is a complex lesion of coronary artery disease (CAD) with a detection rate of approximately 25% on coronary angiography. CTO patients generally experience poor quality of life and prognosis. This study aims to evaluate the association between the estimated glucose disposal rate (eGDR), a surrogate marker for insulin resistance (IR), and the prognosis of CTO PCI patients, as well as to investigate the potential role of the systemic immune-inflammation index (SII) in this process. Methods: We retrospectively included 1482 non-diabetic patients who underwent successful CTO PCI at Anzhen Hospital between January 2018 and December 2021. The primary endpoint was major adverse cardiovascular events (MACEs). Clinical characteristics, biochemical markers, and interventional records were collected, and the eGDR and SII were calculated. Cox regression, restricted cubic splines (RCSs), receiver operating characteristic (ROC) analysis, and Kaplan–Meier curves were used to assess associations. Results: MACEs occurred in 158 patients (10.67%). Patients with MACEs had lower eGDR and higher SII levels. A high eGDR significantly reduced MACE risk (Q4 vs. Q1: HR 0.06, 95% CI 0.03–0.12), while a high SII increased it (Q4 vs. Q1: HR 3.32, 95% CI 1.78–6.33). The combination of low eGDRs and high SIIs predicted the highest MACE risk (HR 4.36, 95% CI 2.71–6.01). The SII partially mediated the relationship between eGDR and MACEs. Conclusions: A low eGDR and high SII are significant predictors of poor prognosis in non-diabetic CTO PCI patients. Combining the eGDR and the SII provides a comprehensive assessment for better predicting cardiovascular outcomes.

## 1. Introduction

Chronic total occlusion (CTO) is one of the more complex lesions of coronary artery disease (CAD), with an estimated detection rate of approximately 25% on coronary angiography [1,2]. Patients with CTO generally experience a poor quality of life and prognosis, making the management of this condition a pressing clinical issue [3]. However, the high procedural difficulty, high complication rates, and prolonged procedure times, coupled with the ongoing debate over the optimal treatment strategy between percutaneous coronary intervention (PCI) and medical therapy, have posed significant challenges [2,4]. Therefore, it is crucial to identify high-risk factors that influence the prognosis of patients undergoing CTO PCI.

Diabetes mellitus is a well-established risk factor for cardiovascular disease. However, in clinical practice, we have observed that some CTO patients do not meet the diagnostic criteria for diabetes but do exhibit insulin resistance (IR). Research on this subgroup of CTO patients is limited. IR is a pathophysiological state characterized by the decreased responsiveness of target organs or tissues to insulin [5], impaired glucose utilization [6], and consequent endothelial dysfunction and exacerbated inflammation [7,8,9]. While there are several methods to evaluate IR, their clinical applicability is limited due to their time-consuming and complex nature [10]. Previous studies have suggested that the estimated glucose disposal rate (eGDR) may be a reliable surrogate marker for IR. This parameter has shown significant heterogeneity between diabetic and non-diabetic populations, with the non-diabetic group being more sensitive to eGDR [11]. Additionally, the inflammatory response is also involved in the development and progression of cardiovascular disease (CVD) and is considered an independent risk factor for predicting patient outcomes [12]. The systemic immune-inflammation index (SII) is a novel inflammatory parameter, and its association with cardiovascular events in patients undergoing PCI has been scarcely studied. Furthermore, the prognostic value of eGDR in CTO PCI patients and the potential role of systemic inflammatory response in this process remain unclear.

To address these gaps, the present study consecutively enrolled 1482 CTO PCI patients with a median follow-up of 36 months, aiming to evaluate the association between the eGDR and the prognosis of CTO PCI patients, as well as to investigate whether the SII mediates this relationship.

## 2. Methods

### 2.1. Study Population and Follow-Up

**Figure 1:**
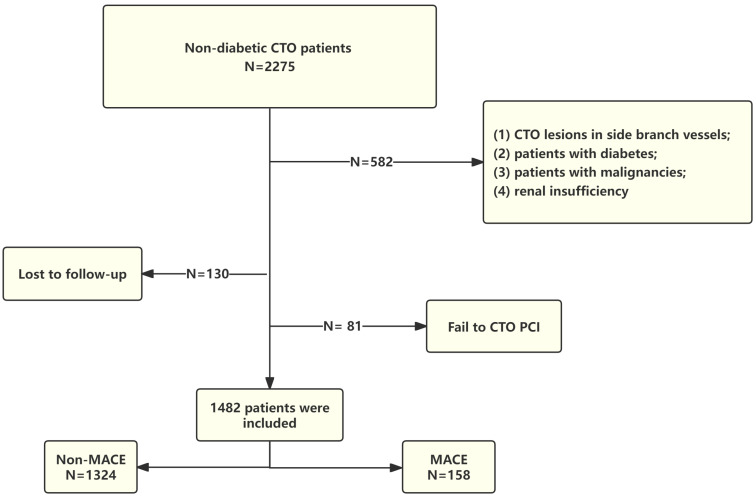
Flow chat of patient screening. CTO, chronic total occlusion; PCI, percutaneous coronary intervention; MACE, major adverse cardiovascular event.

We retrospectively included patients who underwent successful PCI for CTO at Anzhen Hospital from January 2018 to December 2021. CTO was defined as a coronary artery completely occluded for more than three months, involving the left anterior descending artery (LAD), left circumflex artery (LCX), or right coronary artery (RCA). Complete occlusion was characterized by the absence of antegrade flow on coronary angiography, indicated by a Thrombolysis in Myocardial Infarction (TIMI) flow grade of 0 [2]. Successful CTO-PCI was defined as achieving residual stenosis < 30% with a TIMI flow grade ≥ 2 [13]. Exclusion criteria were as follows: (1) CTO lesions in side branch vessels; (2) patients with a history of diabetes, on antidiabetic treatment, or diagnosed with diabetes based on fasting plasma glucose (FPG) ≥ 7.0 mmol/L, HbA1c ≥ 6.5%, or 2 h plasma glucose ≥ 11.1 mmol/L from an oral glucose tolerance test (OGTT) [14]; (3) patients with malignancies; (4) renal insufficiency defined as an estimated glomerular filtration rate (eGFR) < 60 mL/min/1.73 m^2^ [15]. A detailed flow chart is shown in Figure 1. Informed consent was waived due to the retrospective character of the study. However, It has obtained approval from the Human Research Ethics Committee of Beijing Anzhen Hospital, Capital Medical University, Beijing, China (Approval No. 2022177X).

Post-discharge, participants were followed up every 12 months until December 2021. Endpoints were determined through a review of medical records, telephone interviews, and WeChat communications with patients or their families. The primary endpoint was major adverse cardiovascular events (MACEs), including all-cause death, myocardial infarction (MI), and revascularization beyond three months post-discharge. MI was defined by the following: (1) elevated serum cardiac biomarkers (primarily troponin cTn); (2) accompanied by at least one of the following clinical indicators: (a) ischemic symptoms; (b) new ischemic ECG changes; (c) development of pathological Q waves on ECG; (d) imaging evidence of new viable myocardium loss or new regional wall motion abnormalities; (e) coronary angiography or autopsy evidence of an intracoronary thrombus [16]. Coronary artery disease (CAD) lesions were defined as ≥50% stenosis on angiography or ≥70% stenosis on angiography.

### 2.2. Data Collection

Clinical characteristics, biochemical markers, interventional procedure records, and discharge medication information were collected from the hospital’s electronic medical record system. Clinical characteristics included age, sex, smoking status, BMI, and waist circumference (WC). Comorbidities included hypertension, defined as systolic blood pressure ≥ 140 mmHg, diastolic blood pressure ≥ 90 mmHg, or use of an antihypertensive treatment [17]; based on the 2019 European Society of Cardiology/European Society of Atherosclerosis (ESC/EAS) Guidelines for the Management of Dyslipidemia, since all patients in our study were patients with ASCVD, we classified patients with LDL-C greater than 55 mg/dL (1.4 mmol/L) as having dyslipidemia [18]. Relevant medical history was taken, including previous revascularization and MI. Biochemical markers included TG, TC, LDL-C, HDL-C, fasting blood glucose (FBG), glycated hemoglobin (HbA1c), neutrophil (neu) count, lymphocyte (lym) count, and platelet count. The eGDR was calculated as 21.158 − (0.09 × WC) − (3.407 × hypertension) − (0.551 × HbA1c) [19], and the SII was calculated as PLT × (Neu/Lym) [20]. Angiographic information, including the location of CTO lesions (LAD, RCA, LCX, and left main coronary artery (LM)) and the presence of multivessel disease, was obtained from interventional procedure records. Medication information included post-discharge use of antiplatelet, lipid-lowering, and antihypertensive drugs.

### 2.3. Statistical Analysis

Continuous variables are presented as mean ± standard deviation for normally distributed data or median (interquartile range) for non-normal distributions. Categorical variables are displayed as frequency (percentage). Group differences were evaluated using the Chi-square test or Fisher’s exact test, depending on the context. To explore the relationship between the eGDR, the SII, and prognosis in CTO-PCI patients, three Cox regression models were fitted for the eGDR and SII, respectively. Model 1 was adjusted for age, sex, smoking, dyslipidemia, hypertension, and previous MI, previous revascularization; Model 2 was additionally adjusted for CTO lesion characteristics (location and multivessel disease); and Model 3 was further adjusted for medication use (dual antiplatelet therapy, statins, and antidiabetic drugs at discharge) and biochemical indicators (hs-CRP, TG, and HbA1c). Restricted cubic spline (RCS) functions were used to test potential nonlinear relationships between the eGDR, the SII, and MACEs. Receiver operating characteristic (ROC) curve analysis was performed to calculate the area under the curve (AUC) and assess the prognostic value of the eGDR, the SII, and their combination in CTO patients. The optimal cutoff values for the eGDR and SII were determined using ROC curve analysis. Kaplan–Meier curves were used to estimate the cumulative incidence of clinical events, and differences were assessed using the log-rank test. Additionally, mediation analysis was performed to explore whether systemic immune inflammation mediates the effect of insulin resistance on patient prognosis. In addition to the above analysis, we performed subgroup analyses to investigate the association of the eGDR and SII with MACEs based on age (≥60 years), gender, cardiac function (defined by left ventricular ejection fraction (LVEF) > 35%), CTO lesion location, and the completeness of revascularization. All statistical analyses were conducted using R version 4.2.3. A two-sided *p*-value < 0.05 was considered statistically significant.

## 3. Results

### 3.1. Baseline Characteristics

Initially, 2275 patients were included in the study. Of these, 545 patients had diabetes, 37 patients did not meet other inclusion criteria, 81 had unsuccessful procedures, and 130 were lost to follow-up, resulting in their exclusion. Ultimately, 1482 patients were included in the analysis. MACEs occurred in 158 patients (10.67%). Table 1 provides a comparison of baseline data between the groups, stratified by the occurrence of outcome events. Compared with patients without MACEs, those who experienced MACEs had distinct clinical and laboratory characteristics. Clinically, MACE patients were more likely to have hypertension and lower LVEF. Laboratory findings indicated that MACE patients had a lower eGDR and higher SII. Regarding CTO lesions, patients with MACEs were more likely to have lesions located in the LAD.

### 3.2. Impact of eGDR and SII on CTO PCI Patients

**Figure 2:**
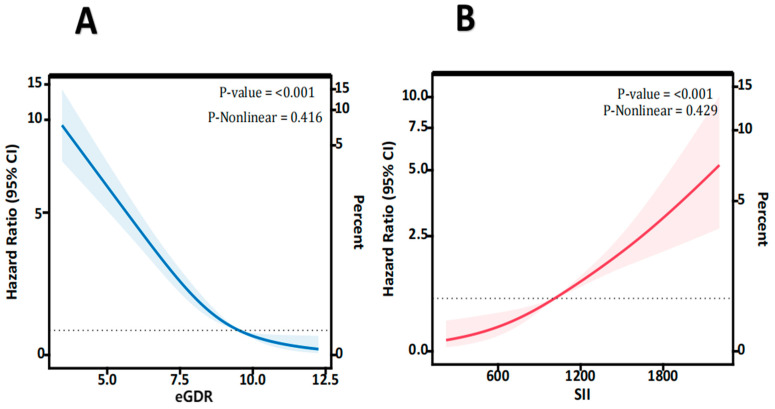
(**A**) The eGDR of Restricted cubic spline curves for CTO-PCI patients; (**B**) The SII of Restricted cubic spline curves for CTO-PCI patients. The dashed lines means HR = 1. CTO, chronic total occlusion; PCI, percutaneous coronary intervention; eGDR, estimated glucose disposal rate; SII, systemic immune-inflammation index.

**Figure 3:**
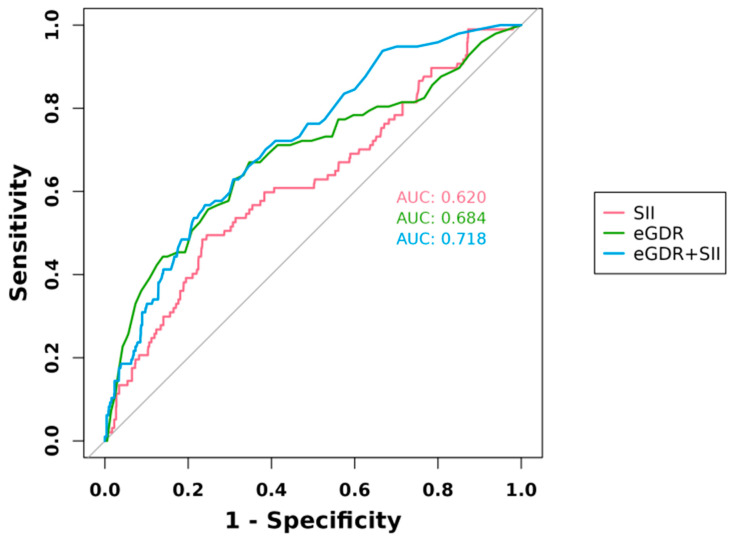
ROC curves for the use of the eGDR, the SII, and their combination in predicting the MACEs of CTO patients. Optimal cutoff: eGDR, 7.10; SII, 710. ROC, receiver operating characteristic; AUC, area under the curve; eGDR, estimated glucose disposal rate; SII, systemic immune-inflammation index.

The association between the eGDR and MACEs is shown in Table 2. Patients with a higher eGDR had a significantly lower risk of MACEs compared with those with lower eGDRs (Q4 vs. Q1: HR 0.06, 95% CI 0.03–0.12). When the eGDR was treated as a continuous variable, each unit increase in the eGDR was associated with a 55% reduction in the risk of MACEs (Table 2). The RCS curve indicated a negative linear relationship between the eGDR and MACEs (nonlinearity *p* = 0.416) (Figure 2A). For predicting MACEs, the ROC curve showed that the AUC, sensitivity, and specificity of the eGDR were 0.684 (95% CI: 0.582–0.708), 78.9 %, and 56.2% (Figure 3). The optimal cutoff value for the eGDR was 7.1. Similarly, patients with higher SII levels had a significantly increased risk of MACEs compared with those with lower SII levels (Q4 vs. Q1: HR 3.32, 95% CI 1.78–6.33). Additionally, each unit increase in SII was associated with a 64% increase in the risk of MACEs (Table 2). The RCS curve also demonstrated a positive linear relationship between the SII and MACEs (nonlinearity *p* = 0.429) (Figure 2B). The AUC, sensitivity, and specificity for MACE prediction using the SII were 0.620 (95% CI 0.534–0.706), 77.3%, and 54.8%, with an optimal cutoff value of 710 for the SII (Figure 3).

### 3.3. Impact of Combined eGDR and SII on Prognosis in CTO PCI

**Figure 4:**
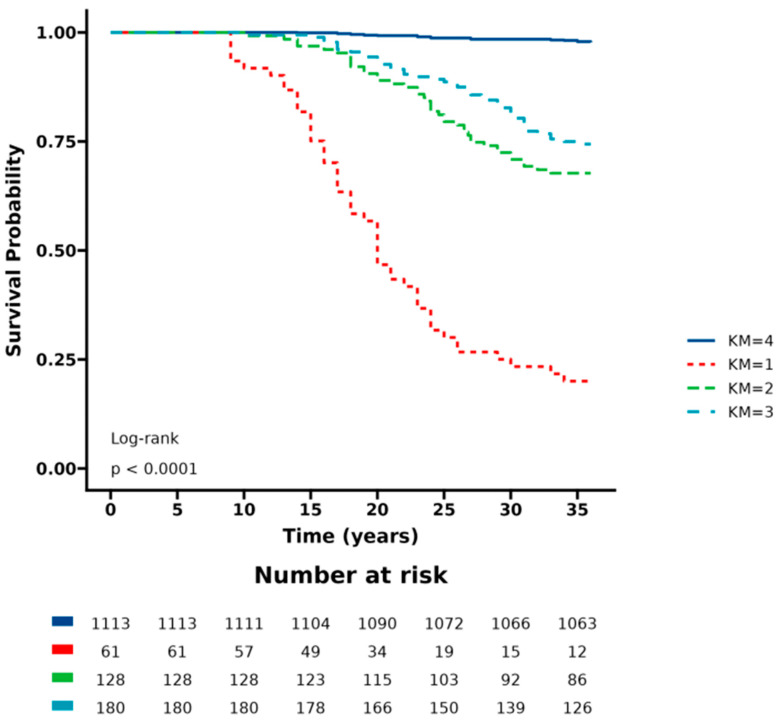
Kaplan–Meier curves for the cumulative incidence of MACEs in patients grouped by the combination of eGDR and SII. KM1: Low eGDR and high SII; KM2: high eGDR and high SII; KM3: low eGDR and low SII; KM4: high eGDR and low SII. eGDR, estimated glucose disposal rate; SII, systemic immune-inflammation index.

In our analysis, we found that patients with a low eGDR index and a high SII had a significantly higher risk of MACEs compared with those with a high eGDR index and a low SII (HR 4.36, 95% CI 2.71–6.01) (Table 3). The ROC curve for the TyG index-SHR combination showed an AUC for MACEs of 0.718 (95% CI 0.619–0.819) (Figure 3). The Kaplan–Meier curve demonstrated that CTO-PCI patients with a low eGDR and a high SII had the highest risk of MACEs during the follow-up period (Figure 4).

### 3.4. Subgroup Analysis

In addition to the above analysis, we performed subgroup analyses to investigate the association of the eGDR and SII with MACEs based on age (≥60 years), gender, cardiac function (defined by ejection fraction > 35%), CTO lesion location, and the completeness of revascularization. The results were consistent with the main findings, showing no significant interaction effects across all subgroups (Table 4).

### 3.5. Mediation Analyses

**Figure 5:**
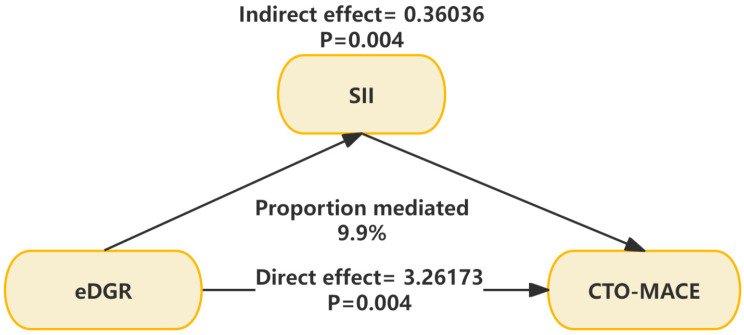
The mediating role of SII in the relationship between eGDR and MACEs. eGDR, estimated glucose disposal rate; SII, systemic immune-inflammation index; MACE, major adverse cardiovascular event.

Furthermore, a mediation analysis was performed to investigate the mediating function of the SII in the correlation between the eGDR and MACEs, as shown in Figure 5 and Table 5. The total effect coefficient for eGDR for the survival data was 3.62209 (95% CI: 3.33176, 4.12312), with a significant *p*-value of 0.004. The indirect effect coefficient mediated by the SII was 0.36036 (95% CI: 0.25529, 0.65750), with a *p*-value of 0.004. The direct effect coefficient was 3.26173 (95% CI: 2.90567, 3.66547), with a *p*-value of 0.004. The proportion of the effect mediated by the SII was 9.9% (95% CI: 7.1, 17.3).

## 4. Discussion

This study is a large-scale, retrospective cohort study investigating the relationship between the eGDR and SII with the prognosis of patients undergoing PCI for CTO. This study is the first to combine the eGDR index with the SII to explore their joint predictive value for adverse outcomes in CTO patients after PCI. The results indicate that patients with a low eGDR index and a high SII have a higher risk of MACEs. The ROC curve analysis shows that the AUC for predicting MACEs using a combination of the eGDR and SII is greater than that of either index alone. We hypothesize that this phenomenon occurs because the eGDR index primarily assesses the degree of insulin resistance, while the SII reflects factors such as inflammatory burden, thereby providing a complementary effect. For predicting MACEs, the ROC curve showed that the AUC, sensitivity, and specificity for the eGDR were 0.684 (95% CI: 0.582–0.708), 78.9%, and 56.2%, respectively. In contrast, the AUC, sensitivity, and specificity for MACE prediction using the SII were 0.620 (95% CI: 0.534–0.706), 77.3%, and 54.8%, respectively. Additionally, the SII mediates the relationship between the eGDR and MACEs.

Previous studies have confirmed that for patients with arteriosclerotic cardiovascular disease (ASCVD), blood glucose levels significantly affect the outcome of intravascular therapy [21]. However, there are still few reports about IR. IR plays a crucial role in the development and progression of CVD [22]. Several mechanisms have been elucidated: IR promotes the progression of atherosclerosis and enhances the oxidation of LDL, and glycated apolipoproteins are more prone to oxidation [23]. Oxidized lipoproteins or apolipoproteins directly inhibit vascular smooth muscle relaxation and stimulate smooth muscle cell proliferation [24]. Insulin also affects thrombosis and platelet aggregation, potentially increasing prothrombotic tendencies by reducing circulating hemostatic markers. Moreover, insulin influences the production and responsiveness of substances related to vascular tone, stimulating the sympathetic nervous system and increasing plasma norepinephrine levels [25]. IR is associated with heightened sensitivity to angiotensin and a 40–50% reduction in nitric-oxide-mediated vasodilation caused by the endothelium, leading to matrix protein deposition and fibrosis and ultimately decreasing vascular relaxation function. IR can also cause renal sodium and water retention, increasing blood volume. In normal individuals, intravenous insulin reduces renal sodium excretion by 50%, a physiological effect absent in IR patients [26,27]. Given the importance of IR, several indices have been developed to assess it, such as the hyperinsulinemic–euglycemic clamp technique and the triglyceride–glucose index. However, these indices often have limitations regarding convenience, specificity, and sensitivity in clinical use [22,26]. Thus, our study employs a novel index, the eGDR, to evaluate IR in patients with coronary artery occlusion. Previous studies have shown significant heterogeneity in the eGDR among diabetic and non-diabetic populations, with the eGDR being more sensitive in non-diabetic individuals [10]. While earlier studies have attempted to use the eGDR to assess CVD prognosis, the predictive ability of the eGDR in non-diabetic CVD patients remains unclear due to the confounding effects of diabetes [11]. Therefore, this study focuses on non-diabetic CTO PCI patients to clarify the impact of IR on their prognosis. Recent clinical research has confirmed that eGDR levels significantly affect the prognosis of non-diabetic CVD patients, with each 1.0 standard deviation increase in the eGDR reducing CVD risk by 17% (HR: 0.83, 95% CI: 0.78–0.89), consistent with our findings [26].

Beyond these mechanisms, IR may also stimulate systemic inflammatory responses, further impairing endothelial function [27]. The SII is a novel, stable inflammatory marker that reflects local immune responses and systemic inflammation [28]. The SII is calculated as platelet count × neutrophil count/lymphocyte count, providing a comprehensive reflection of the inflammatory state compared with single inflammatory markers [29]. Initially used to predict cancer prognosis, SII has also been associated with the development of cerebrovascular diseases. A recent study found that high SII levels are independently associated with an increased risk of cardiac events in patients undergoing PCI [29]. In our study, we determined the optimal SII cutoff (≥710) for predicting MACEs in CTO PCI patients. Recent studies have confirmed that high SII levels correlate with lower collateral circulation scores in CTO patients, possibly because inflammatory cells and factors affect the development of coronary collaterals [30]. Patients with better collateral circulation have improved protection, providing blood supply during major coronary occlusions, reducing myocardial ischemia, and improving myocardial function. In addition to affecting collateral circulation, inflammation is a crucial factor in the development of in-stent restenosis (ISR) [31]. During early post-stent placement, mechanical injuries related to the stent can cause endothelial cell rupture and dysfunction or plaque rupture, triggering inflammatory responses and platelet activation. In later stages, smooth muscle cell proliferation and migration and extracellular matrix production can lead to neointimal hyperplasia and atherosclerosis. Inflammatory responses contribute to neointimal hyperplasia and atherosclerosis formation, promoting ISR development and affecting post-PCI prognosis [32].

## 5. Limitations

Although this study included a large consecutive sample, several limitations remain. First, as a single-center retrospective study, some biases are inevitable. Additionally, the 130 patients who were excluded from the study due to loss to follow-up may have characteristics and outcomes that differ from those included, which could lead to selection bias. Future prospective multi-center studies are needed to confirm the robustness of our conclusions. Second, the eGDR and SII are time-dependent variables, and due to follow-up constraints, we could not observe their trajectory over time. Our next step is to collaborate more closely with community health centers to monitor the impact of these indices’ trajectories on the prognosis of CTO PCI patients. Third, previous studies have confirmed that life’s essential 8 (LE8) factors are important independent risk factors for CVD patients [33], but our study did not include LE8 assessments. Fourth, we did not incorporate coronary functional characteristics, such as coronary blood flow and flow reserve. The impact of insulin resistance and systemic inflammation on coronary function warrants further investigation.

## 6. Conclusions

In conclusion, our study highlights the importance of combining the eGDR and SII to better predict the prognosis of non-diabetic patients undergoing CTO PCI, offering a more comprehensive assessment of insulin resistance and systemic inflammation’s impact on cardiovascular outcomes.

## Figures and Tables

**Table 1:** Baseline characteristics of participants.

	Non-MACE (n = 1324)	MACE (n = 158)	*p*-Value
Age, years	59.9 ± 10.6	61.4 ± 10.7	0.442
Male, n (%)	1077 (81.4)	129 (82.1)	0.790
BMI, kg/m^2^	27.4 ± 5.6	29.2 ± 6.8	0.021
WC, cm	90 ± 14	94 ± 15	<0.001
Smoking, n (%)	442 (33.4)	51 (32.2)	0.340
Hypertension, n (%)	932 (70.41)	120 (76.07)	0.009
Dyslipidemia, n (%)	1116 (84.29)	135 (85.40)	0.844
LVEF, %	60.1 ± 7.4	54.7 ± 7.4	0.023
Prior MI, n (%)	392 (29.6)	48 (30.5)	0.124
Prior revascularization, n (%)	456 (34.42)	51 (32.3)	0.366
Laboratory tests			
Platelet, ×10^9^/L	235 ± 56	275 ± 75	0.001
Lymphocyte, 10^3^/µL	2.04 ± 0.60	1.94 ± 0.73	0.124
Neutrophils, 10^3^/µL	3.66 ± 1.34	5.21 ± 1.99	<0.001
HbA1c, %	5.41 ± 0.48	5.55 ± 0.40	<0.001
FBG, mg/dL	98 ± 18	101 ± 12	<0.001
TC, mg/dL	195 ± 41	198 ± 40	0.177
TG, mg/dL	120 ± 97	130 ± 73	0.062
LDL-C, mg/dL	114 ± 36	119 ± 35	0.077
HDL-C, mg/dL	55 ± 16	52 ± 16	0.191
eGDR (mg/kg/min)	9.64 (8.55, 10.61)	6.72 (5.02, 8.72)	<0.001
Hs-CRP, mmol/L	2.8 ± 3.3	3.4 ± 3.5	0.006
SII	409 (305, 562)	810 (495, 986)	<0.001
Intervention treatment, n (%)			
LM	25 (1.9)	0	0.124
LAD	419 (31.7)	55 (35.6)	0.042
LCX	184 (13.9)	22 (13.8)	0.466
RCA	695 (52.5)	81 (50.6)	0.225
Multivessel disease	1162 (87.8)	147 (93.5)	0.051
Complete revascularization	1247 (84.23)	125 (79.07)	0.028
Medication at discharge, n (%)			
DAPT	1322 (99.9)	157 (99.4)	0.580
Antihypertensive	864 (65.25)	100 (63.29)	0.209
Statin	1283 (96.9)	152 (96.3)	0.676

eGDR, estimated glucose disposal rate; SII, systemic immune-inflammation index; MACE, major adverse cardiovascular event; BMI, body mass index; WC, waist circumference; LVEF, left ventricular ejection fraction; MI, myocardial infarction; TC, total cholesterol; HDL-C, high-density lipoprotein cholesterol; LDL-C, low-density lipoprotein cholesterol; HbA1c, glycosylated hemoglobin A1c; TG, triglyceride; FBG, fasting blood glucose; LAD, left anterior descending artery; LCX, left circumflex artery; RCA, right coronary artery; LM, left main; DAPT, dual antiplatelet therapy.

**Table 2:** Cox regression models for the association of the eGDR and SII with MACEs.

Characteristic	Model 1			Model 2			Model 3		
	HR	95% CI	*p*-Value	HR	95% CI	*p*-Value	HR	95% CI	*p*-Value
eGDR (continuous)	0.47	0.42, 0.52	<0.001	0.49	0.44, 0.55	<0.001	0.55	0.51, 0.60	<0.001
eGDR									
Q1 (<8.08)	—	—		—	—		—	—	
Q2 [8.08, 9.51)	0.11	0.06, 0.18	<0.001	0.13	0.07, 0.22	<0.001	0.19	0.12, 0.30	<0.001
Q3 [9.51, 10.5)	0.09	0.05, 0.15	<0.001	0.11	0.06, 0.20	<0.001	0.15	0.09, 0.25	<0.001
Q4 (≥10.5)	0.02	0.01, 0.07	<0.001	0.04	0.01, 0.10	<0.001	0.06	0.03, 0.12	<0.001
SII (continuous)	1.57	1.52, 1.72	<0.001	1.59	1.54, 1.65	<0.001	1.64	1.57, 1.70	<0.001
SII									
Q1 (<316)	—	—		—	—		—	—	
Q2 [316, 431)	1.39	0.72, 3.01	0.224	1.49	0.77, 3.18	0.211	1.53	0.81, 3.20	0.181
Q3 [431, 597)	2.18	0.87, 3.21	0.101	2.21	0.90, 3.24	0.092	2.31	0.95, 3.35	0.059
Q4 (≥597)	3.02	1.65, 5.52	<0.001	3.20	1.72, 5.93	<0.001	3.32	1.78, 6.33	<0.001

Model 1 was adjusted for age, sex, smoking, dyslipidemia, hypertension, and previous MI, previous revascularization; Model 2 was additionally adjusted for CTO lesion characteristics (location and multivessel disease); and Model 3 was further adjusted for medication use (DAPT, statins, and antidiabetic drugs at discharge) and biochemical indicators (hs-CRP, TG, and HbA1c). eGDR, estimated glucose disposal rate; SII, systemic immune-inflammation index; MACE, major adverse cardiovascular event; MI, myocardial infarction; TG, triglyceride; HbA1c, glycosylated hemoglobin A1c; DAPT, dual antiplatelet therapy.

**Table 3:** Cox regression models for the association of the combination of eGDR and SII with MACEs.

Characteristic	Model 1			Model 2			Model 3		
	HR	95% CI	*p*-Value	HR	95% CI	*p*-Value	HR	95% CI	*p*-Value
High eGDR and low SII	-	-	-	-	-	-	-	-	-
Low eGDR and low SII	1.27	(0.78–2.01)	0.211	1.31	(0.82–2.16)	0.171	1.54	(0.94–2.14)	0.103
High eGDR and High SII	1.03	(0.54–1.57)	0.501	1.07	(0.63–1.77)	0.371	1.39	(0.76–2.01)	0.229
Low eGDR and High SII	3.53	(2.29–4.75)	<0.001	3.74	(2.36–5.12)	<0.001	4.36	(2.71–6.01)	<0.001

Model 1 was adjusted for age, sex, smoking, dyslipidemia, hypertension, previous MI, and previous revascularization; Model 2 was additionally adjusted for CTO lesion characteristics (location and multivessel disease); and Model 3 was further adjusted for medication use (DAPT, statins, and antidiabetic drugs at discharge) and biochemical indicators (hs-CRP, TG, and HbA1c). eGDR, estimated glucose disposal rate; SII, systemic immune-inflammation index; MACE, major adverse cardiovascular event; MI, myocardial infarction; TG, triglyceride; HbA1c, glycosylated hemoglobin A1c; DAPT, dual antiplatelet therapy.

**Table 4:** Subgroup analyses of the associations between eGDR, SII, and the risk of MACEs.

Subgroup	eGDR	*p* for Interaction	SII	*p* for Interaction
	HR 95% CI		HR 95% CI	
Age (years)		0.133		0.311
<60	0.65 (0.55–0.76)		1.92 (1.42–2.05)	
≥60	0.75 (0.65–0.87)	0.081	2.17 (1.13–4.19)	
Sex				0.523
Male	0.83 (0.75–0.93)		2.31 (1.80–2.94)	
Female	0.70 (0.62–0.78)	0.233	1.87 (1.57–2.05)	
LVEF				0.801
<35	0.89 (0.79–1.01)		7.01 (1.90–12.56)	
≥35	0.82 (0.75–0.91)		2.37 (1.37–5.08)	
CTO		0.647		0.497
LAD	0.90 (0.82–0.98)		2.86 (1.45–4.54)	
RCA	0.66 (0.57–0.77)		2.11 (1.38–3.00)	
LCX	0.71 (0.61–0.82)		2.9 (1.01–4.56)	
Complete revascularization		0.079		0.569
Yes	0.71 (0.61–0.82)		1.75 (1.01–3.08)	
No	0.91 (0.82–1.02)		8.88 (5.28–14.75)	

eGDR, estimated glucose disposal rate; SII, systemic immune-inflammation index; MACE, major adverse cardiovascular event; LVEF, left ventricular ejection fraction; LAD, left anterior descending artery; LCX, left circumflex artery; RCA, right coronary artery.

**Table 5:** The mediating role of SII in the relationship between eGDR and MACEs. eGDR, estimated glucose disposal rate; SII, systemic immune-inflammation index; MACE, major adverse cardiovascular event.

Independent Variable	Mediator	Total Effect		Indirect Effect		Direct Effect		Proportion Mediated, % (95% CI)
		Coefficient (95% CI)	*p*-Value	Coefficient (95% CI)	*p*-Value	Coefficient (95% CI)	*p*-Value	
eGDR	SII	3.62209 (3.33176, 4.12312)	0.004	0.36036 (0.25529, 0.65750)	0.004	3.26173 (2.90567, 3.66547)	0.004	9.9 (7.1, 17.3)

## Data Availability

The datasets used and/or analyzed during the current study are available from the corresponding author upon reasonable request.
